# An integrative approach to inferring biologically meaningful gene modules

**DOI:** 10.1186/1752-0509-5-117

**Published:** 2011-07-26

**Authors:** Ji-Hoon Cho, Kai Wang, David J Galas

**Affiliations:** 1 Institute for Systems Biology, 401 N Terry Avenue, Seattle, WA 98109, USA

## Abstract

**Background:**

The ability to construct biologically meaningful gene networks and modules is critical for contemporary systems biology. Though recent studies have demonstrated the power of using gene modules to shed light on the functioning of complex biological systems, most modules in these networks have shown little association with meaningful biological function. We have devised a method which directly incorporates gene ontology (GO) annotation in construction of gene modules in order to gain better functional association.

**Results:**

We have devised a method, Semantic Similarity-Integrated approach for Modularization (SSIM) that integrates various gene-gene pairwise similarity values, including information obtained from gene expression, protein-protein interactions and GO annotations, in the construction of modules using affinity propagation clustering. We demonstrated the performance of the proposed method using data from two complex biological responses: 1. the osmotic shock response in *Saccharomyces cerevisiae*, and 2. the prion-induced pathogenic mouse model. In comparison with two previously reported algorithms, modules identified by SSIM showed significantly stronger association with biological functions.

**Conclusions:**

The incorporation of semantic similarity based on GO annotation with gene expression and protein-protein interaction data can greatly enhance the functional relevance of inferred gene modules. In addition, the SSIM approach can also reveal the hierarchical structure of gene modules to gain a broader functional view of the biological system. Hence, the proposed method can facilitate comprehensive and in-depth analysis of high throughput experimental data at the gene network level.

## Background

High throughput technologies to accurately profile the transcriptome have been the major thrust of modern systems biology [1,2]. Based on the premise that co-expressed genes and/or closely interconnected proteins are more likely to be related to a particular biological function, researchers have made an effort to identify groups of genes, called modules [3], to gain a better understanding of the biological system of interest. Early attempts in building such gene modules depended primarily on the co-expression property of genes [4-8]. Combining gene expression with protein-protein interaction data greatly expanded the modules and in some cases enhanced the functional association of modules [9-15]. Although gene expression and protein interaction information have been used in constructing gene modules, these approaches have not taken advantage of the vast amount of knowledge accumulated about gene annotation/function. Therefore, these approaches would not be expected to be maximally effective in constructing modules with strong association to biological functions [16,17].

Recently, the concept of semantic similarity, which was developed for and used in the field of natural language processing [18-20] has been applied to analyze gene ontology terms and used to predict and confirm protein functions and interactions [21-24]. Wang et al. [25] developed a Gene Ontology (GO) [26] structure-based measure to quantify semantic similarity between individual terms as well as genes and showed the advantage of using semantic similarity in organizing complex biological terms.

We postulated that the direct incorporation of semantic similarity based on GO annotation could significantly enhance the construction of biologically meaningful gene modules which have strong associations with known biological functions. We have therefore developed SSIM (Semantic Similarity-Integrated approach for Modularization), to integrate various gene-gene pairwise relationships including similarity measures based on GO biological process (BP) annotation of genes, gene expression pattern, and protein-protein interaction information. The integrated information is then used to group genes into modules using affinity propagation [27]. Affinity propagation is a clustering method that does not use pre-selected centers for clustering, instead it generates exemplars that best represent a group of data points, in this case genes, by considering all similarities between pairs of data points and testing all data points as potential exemplars. A group of data points that have the same exemplar can then be considered to be in a cluster. The modules generated by SSIM are found to be more significantly and specifically associated with biological functions than the results obtained from two methods, Module Analysis *via *Topology of Interactions and Similarity Sets (MATISSE) [12] and Interaction Component Models for Gene modules (ICMg) [14].

## Results and discussion

MATISSE was originally evaluated using the expression information of 1990 osmotic stress-associated genes in yeast and ~69,000 yeast protein interactions. ICMg was tested with a slightly smaller set of osmotic stress-associated genes and using a different set of protein interaction data (see methods). MATISSE also adapted a strategy to generate connected modules by including information from genes that are not in the original selected gene set, referred to back nodes [12]. ICMg focused on the information within the set of selected genes only.

To compare and evaluate the performance of SSIM, we analyzed the same datasets used in MATISSE and ICMg studies (MATISSE dataset and ICMg dataset). Since MATISSE and ICMg used probabilistic approaches to optimize the construction of gene modules, the inferred connections and number of modules could vary in each run. To obtain the number of modules for comparison, MATISSE was executed 20 times as described [14], which yielded the median number of gene modules of 24 for both datasets. ICMg was then also executed 20 times with a fixed number of modules at 24. For SSIM, we adjusted the preference value to obtain the same number of modules (see methods).

### Semantic similarity-integrated approach for modularization (SSIM) generates functionally relevant gene modules

GO enrichment analysis with statistical testing such as Fisher exact test was the most commonly used approach to evaluate the functional association of individual gene modules. A low *p*-value between a gene module and a GO term would imply a strong association of the module with the specific biological function represented by the term. Since SSIM integrates semantic similarity of GO BP (biological process) terms in gene module construction, it is expected to have a better GO enrichment performance compared to other methods. Therefore a different annotation scheme, MIPS FunCat [28], which is independent of GO, was also used to evaluate the functional associations of gene modules.

**Figure 1:**
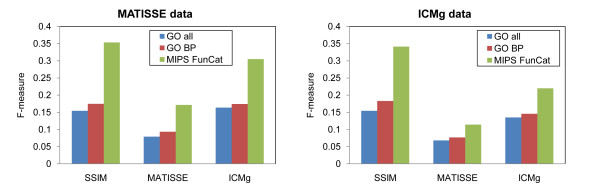
**Functional enrichment analysis results**. The functional enrichment performance of each method was evaluated using three different annotation datasets, all GO terms, GO BP terms and MIPS FunCat terms. The results were summarised using *F*-measure (Y-axis, see text). Results for MATISSE and ICMg were obtained using the mean values of 20 runs.

For a given significance level, the number of modules enriched with at least one annotation term and the number of annotation terms enriched in at least one module are referred as *specificity *and *sensitivity *[13]. For each method, the sensitivity and specificity were calculated and summarized into a measure of functional enrichment, an *F*-measure defined as *F *= 2 × Sensitivity × Specificity/(Sensitivity + Specificity) [13]. Note that the ratio of modules enriched with at least one annotation term (i.e. specificity) might be also expressed as precision. The results from SSIM showed better functional enrichment significance (*F-*measure) for MIPS FunCat annotations (as well as GO and GO BP terms) than other methods (Figure 1 and Additional File 1). ICMg gave comparable results to SSIM, whereas MATISSE showed lower performances in terms of both sensitivity and specificity. This could be due to a constraint on the size of the modules (a default parameter with no more than 100 genes per module was used in this study, but it is adjustable by user) and the addition of back nodes (genes that were not in the initial set of genes but were later included to make connected gene modules) in MATISSE.

To further evaluate the overall functional enrichment performance from the three different methods, semantic similarity between the terms and the shortest paths of the terms to the root (e.g. GO:0008150, biological process) were investigated (see methods). If enriched terms were closely related to each other (coherency) and were far away from the root in GO hierarchy (depth), they may reveal more specific and detailed biological functions. Enriched GO terms associated with the modules from SSIM have higher average semantic coherency, which measures how enriched GO terms are coherent in terms of semantic similarity, and comparable depth relative to other methods (see method and Additional file 2).

### SSIM produces modules with strong functional association and high expression homogeneity

**Figure 2:**
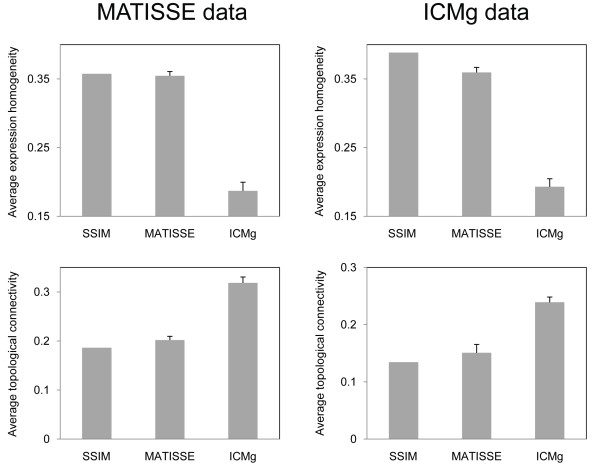
**Expression homogeneity and topological connectivity of modules**. Expression homogeneity and topological connectivity of a module were calculated using average Pearson correlation among genes in the module and average clustering coefficient of the network generated by the genes, respectively. Average expression homogeneity and connectivity over all modules were shown for each method. For MATISSE and ICMg, mean and standard deviation over 20 runs were taken.

We also investigated the homogeneity of gene expression profiles and topological connectivity of modules generated by three different methods using average Pearson correlation as well as average clustering coefficient [29] of genes within the same module. As shown in Figure 2, for both datasets, SSIM and MATISSE yielded modules with similar levels of expression homogeneity and topological connectivity while ICMg produced modules composed of densely connected genes with poorly co-expressed profiles.

This finding also suggests the possibility of using SSIM as a tool to explore gene regulatory networks since genes with similar expression profiles are commonly co-regulated [4,7,30-33]. Notably, the study conducted by Ulitsky and Shamir [12] indicates that modules generated by random sampling of genes with sufficient network connectivity could give favorable topological properties and functional enrichment results, but with much lower expression homogeneity. This implies that significant GO enrichment results for gene modules might be obtained by chance if we just considered topological connectivity of genes; thus, additional criteria such as gene expression homogeneity must be used to ensure the reliability of functional association for gene modules.

**Figure 3:**
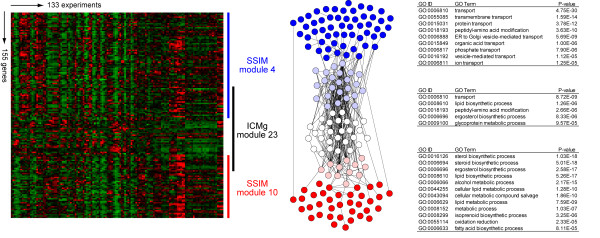
**Comparison between modules identified by ICMg and SSIM**. One of the modules identified by ICMg method (ICMg module) shares a number of genes with two separate modules identified by SSIM (SSIM module A and B). Expression profiles of genes in three modules were shown on the left and protein-protein interaction network was shown in the middle. Genes shared by ICMg and SSIM modules are indicated by pink and light blue nodes, and ones exclusively belong to ICMg, SSIM module A and SSIM module B are depicted by white, red and blue nodes, respectively. In the right panel, enriched GO BP terms (*p *< 1 × 10^-5^) and their uncorrected *p*-values for each module are summarized.

For a given number of modules, the SSIM approach seems to generate gene modules with better functional association and higher correlations in their expression homogeneity. For example, one of the ICMg modules (ICMg module 23) shared a large portion of genes with two modules generated by SSIM (SSIM module 4 and 10) (see methods and Additional File 3, Table S1). The GO terms enriched in the ICMg module 23 implicated two biological functions, "transport" and "lipid biosynthetic process" while in SSIM, the two functions were separated into two different modules, module 4 and 10, with much higher functional association (lower *p*-value) and expression homogeneity (Figure 3 and Additional file 3, Table S2 and S4).

### SSIM can be used to reveal the hierarchical structure of gene modules

**Figure 4:**
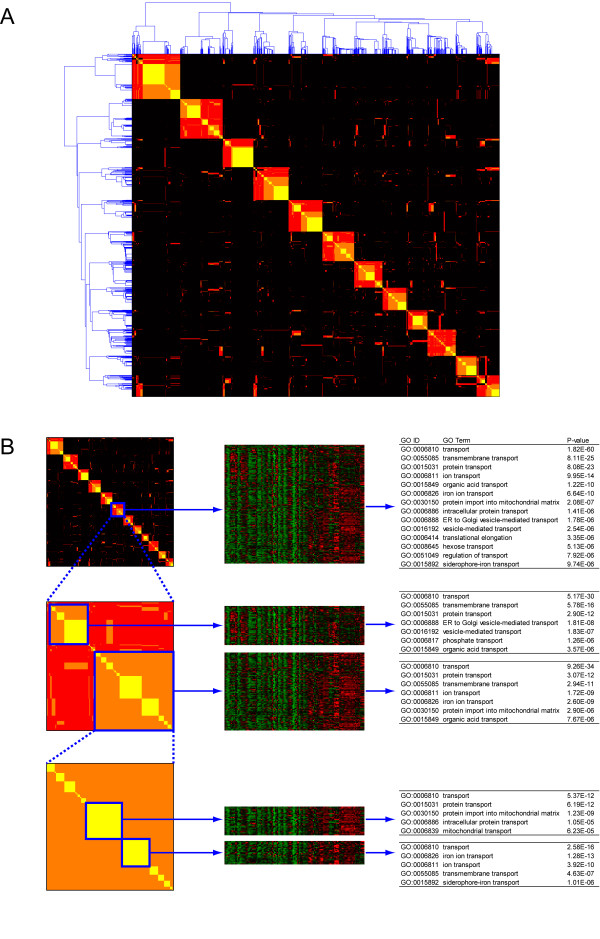
**Hierarchical structure of modules generated by SSIM approach**. Using different preference values, three sets of modules (12, 18 and 37 modules) were obtained (see Additional file 4) and the membership of genes over the three module sets was expressed as a matrix form. When gene *i *and *j *belong to the same module and the membership is conserved in any one, two and three sets, the element of matrix in *i*th row and *j*th column is set to 1 (red, least conserved over the sets of modules), 2 (orange) and 3 (yellow, most conserved), respectively. If two genes are not in the same module, the corresponding element in the matrix has a zero value (black). Hierarchical clustering result of the matrix was shown in (A). Most orange and yellow squares are subsets of red squares along the diagonal, which means that a large module is hierarchically split into several smaller modules according to the change of preference values. (B) As an example, the decomposition of a large module (red square) identified in "12-module set" into smaller modules obtainable in "18-" and "37-module set" is shown with the expression profiles of genes and enriched GO BP terms. A module representing general transport function (12-module set) is stratified into modules specifically related to protein and iron ion transport (37-module set).

To compare the efficiency of constructing gene modules with different algorithms, we have used a fixed number of modules (24 modules in this study). In SSIM, a larger preference value of affinity propagation [27] means that every gene is more likely to be an exemplar of a module (module center), which would produce a large number of modules composed of highly similar genes in terms of integrated similarity and allow us to view the system in detail. A smaller preference value would generate fewer modules which offer a broader, less detailed overview of the system. This implies that SSIM can also be used as a tool to generate a functional hierarchy of gene modules by virtue of using semantic similarity. As an example, we applied the SSIM approach to a MATISSE dataset with a wide range of preference values and chose three sets of modularization results with 12, 18 and 37 modules (see Additional file 4) to illustrate the hierarchical structure of gene module generated by SSIM. Figure 4A shows that large-size modules obtained from a smaller preference value were hierarchically decomposed into smaller modules using a larger preference value. As an example of the hierarchical structure of gene modules generated with SSIM using different preference values, one of the module obtained from the "12-module set" representing various "transport" functions is split into two groups, based on the membership of "18-module set", showing slightly different expression profiles. One of the two groups from the "18-module set" is further divided into two smaller groups based on "37-module set", which further stratify the "transport" function into protein and ion transport (Figure 4B).

### Extension of the module identified by SSIM approach

Suppose that we have information about a set of genes *U *and thereby choose a subset *V *as genes of interest to be used to construct gene modules. The remaining genes (*V^C^*), where *V *∪*V^C ^*= *U *and *V *∩*V^C ^*= *ø*, are generally not considered in constructing gene modules. However, the genes in *V^C ^*might have many interactions with the genes in gene set *V *and potentially provide important information in representing the biological function of the constructed modules. Such information can be incorporated by including these genes in *V^C ^*using a module extension procedure (see methods). As an example, one of the modules identified by SSIM (using the MATISSE dataset) had 72 genes representing functions related to "cell cycle". The addition of 55 neighboring interconnected genes from *V *that were not in the initial gene list *V^C ^*increased the significance level of enriched GO BP terms and the average clustering coefficient from 0.0648 to 0.2934 (see Additional File 5).

### SSIM can be used to reveal biological processes involved in complex disease

The recently described gene networks involved in prion disease model was a significant development in the advance of systems biology [34]. It demonstrated the value of constructing gene networks to reveal biological processes involved in this complex disease. The study analyzed gene expression profiling results from several different experimental conditions and time points for prion disease animal model. A set of roughly 300 differentially expressed genes (DEGs) that were common to different experimental conditions (mouse strains and prion types) was identified from these analyses. These DEGs were first classified by GO annotations, then hand-curated and assigned into different gene networks based on functional enrichment results and prior knowledge of the genes. Additional non-differentially expressed genes were also included to complete and illustrate the functionality of the networks. It was surprising to find that the pathogenesis of prion disease can be largely explained by only four major networks: PrP^Sc ^replication and accumulation, microglial and astrocytic activation, synaptic degeneration, and neuronal cell death [34]. Genes in these four networks are involved in proteolysis and lipid metabolism for PrP^Sc ^replication and accumulation network, immune responses for microglial and astrocytic activation network, mitochondrial dysfunction and apoptosis for neuronal cell death network, and transcription-related function, intracellular signal transduction and ion transport-related function for synaptic degeneration network.

To test the feasibility of using SSIM in complex disease analysis, the same set of genes used in the prion network construction was fed into SSIM. A total of 16 gene modules were obtained and they could largely subsume into the four larger manually generated networks previously described (see Table 1 and Additional File 6). For example, modules 1 and 3 contain a total of 36 genes that are highly associated with lipid metabolism and proteolysis based on GO BP enrichment result, which almost completely recapitulate the key nodes involved in PrP^Sc ^accumulation network. In addition to the modules related directly to the four reported networks, SSIM also suggested processes involved in tissue remodeling such as actin cytoskeleton organization (GO:0030036), angiogenesis (GO:0001525), multicellular organismal development (GO:0007275) and cell differentiation (GO:0030154) might also be involved in the progression of prion disease. We will conduct more detailed network analyses with SSIM using prion disease datasets including the comparison between different incubation times, host strains, and infectious agents, since this preliminary study agrees well with the manually constructed networks.

**Table 1:** Similarity between modules identified by SSIM and four major prion subnetworks

Module	Genes in the module	PrP^Sc ^accumulation	Microglial activation	Neuronal cell death	Synaptic degeneration
1	21	16 (76%)	5 (24%)	2 (10%)	0 (0%)
2	27	7 (26%)	21 (78%)	1 (4%)	1 (4%)
3	15	12 (80%)	0 (0%)	3 (20%)	0 (0%)
4	37	5 (14%)	5 (14%)	10 (27%)	20 (54%)
5	42	16 (38%)	14 (33%)	13 (31%)	4 (10%)
6	33	1 (3%)	1 (3%)	12 (36%)	24 (73%)
7	34	5 (15%)	22 (65%)	9 (26%)	2 (6%)
8	37	6 (16%)	0 (0%)	9 (24%)	24 (65%)
9	49	2 (4%)	7 (14%)	37 (76%)	6 (12%)
10	41	19 (46%)	19 (46%)	8 (20%)	2 (5%)
11	39	7 (18%)	29 (74%)	5 (13%)	4 (10%)
12	43	2 (5%)	13 (30%)	18 (42%)	12 (28%)
13	46	19 (41%)	38 (83%)	2 (4%)	0 (0%)
14	57	5 (9%)	36 (63%)	17 (30%)	7 (12%)
15	67	5 (7%)	2 (3%)	23 (34%)	49 (73%)
16	60	5 (8%)	0 (0%)	2 (3%)	54 (90%)

For each module, the number and the proportion of genes corresponding to prion subnetworks are shown. Since some genes are involved in multiple subnetworks, the row-wise summation of proportions may exceed 100%.

## Conclusions

In this report, we have proposed and demonstrated a new approach, Semantic Similarity-Integrated approach for Modularization (SSIM), for inferring biologically meaningful gene modules. The SSIM method integrates various gene-gene pairwise similarity information obtained from gene expression profiling results, protein interactions and GO annotations to construct gene modules. We showed that gene modules generated by SSIM gave higher specificity with stronger association with biological functions based on the assessment of GO and MIPS annotation terms.

Since SSIM is based on pairwise similarity values of genes, there is room for further improvement by integrating additional quantitative similarity measures. For example, GO semantic similarity of other categories (molecular function (MF) and cellular component (CC)) or newly developed semantic similarity measures [35-37] can also be used. In addition, different clustering algorithms can also be used to enhance the construction of gene modules. The evaluation and comparison between various gene pairwise similarities and clustering methods will be interesting to explore in the future.

We have also demonstrated the ability to use SSIM to uncover the hierarchical structure of gene modules, i.e. hierarchical association and dissociation of modules at different levels of functional detail. This would allow us to gain a systematic understanding of a given biological system. When some genes are excluded from the set of selected genes (often differentially expressed genes) due to marginal expression changes or for other reasons, the module extension procedure adapted in SSIM can incorporate them and increase the functional interpretability of gene modules without sacrificing the statistical significance of functional association of gene modules. Although a similar approach has been used in other studies [12,15], the effect of network extension on functional association of gene modules has not been evaluated carefully in those methods.

Even though tools to construct gene modules have been developed, they have been mainly applied in datasets from relatively simple model organisms [4,5,9,11,13,14]. The SSIM performed well not only with the dataset from yeast, but also with a much more complicated dataset, the prion disease mouse model. Gene modules from SSIM effectively recaptured the manually constructed key networks described in prion disease, and also revealed new processes that might also be involved in the disease development. Our results suggest the SSIM approach can quickly infer gene modules with coherent biological meaning and thereby accelerate systems biology studies in complex diseases.

## Methods

### Reference datasets and methods

We used 133 expression profiles representing the osmotic shock response of *Saccharomyces cerevisiae *in various conditions [38]. Ulitsky and Shamir [12] selected 1990 genes and curated ~69,000 protein interactions for MATISSE. Parkkinen and Kaski [14] used 1771 genes and ~10,000 interactions for ICMg. While 1771 genes are a subset of 1990 genes, ~10,000 interactions are not exactly a subset of ~69,000 interactions since they used a different approach to curate yeast protein interaction data. Both of the datasets were used to compare and evaluate the performance of all three different methods. We used implementations of MATISSE (Java software) and ICMg (R-package) with their default parameters. These datasets (MATISSE data and ICMg data) and implementations were downloaded at http://acgt.cs.tau.ac.il/matisse and http://www.cis.hut.fi/projects/mi/software/ICMg, respectively.

### Similarity measures

Building gene modules can be viewed as the grouping genes of interest according to their pairwise similarity values. Pearson correlation between each pair of genes (gene *i *and gene *j*), was used as expression similarity, *e_ij_*. From the global protein-protein interaction network, pairwise topological similarity (*t_ij_*) was obtained using topological overlap matrix [39] which was initially developed to identify gene modules in *E. Coli *metabolism. It reaches the maximum value of 1 when there is a direct connection between two genes and has the value between 0 and 1 when two genes are not linked but share some direct neighbors. If there is no direct connection between two genes and no direct neighbors shared by them, *t_ij _*has the minimum value of 0. GO semantic similarity between two genes (*g_ij_*) was computed by the metric proposed by Wang et al. [25] which took the characteristics of GO hierarchy into account and overcame drawbacks of previous semantic similarity measures [18-20]. *g_ij _*has the value of 0 for two genes with no similarity and 1 for genes having identical GO annotations. Although GO semantic similarity can be computed for three categories of GO terms, biological process (BP), molecular function (MF) and cellular components (CC), we used the similarity of GO BP terms in this study. An overview of GO semantic similarity can be found in Pesquita et al. [40].

To combine three similarity measures, they were assumed to be independent of each other, which is probably a good first approximation since they were derived from different information sources. First, for expression similarity, the empirical cumulative density function, *F*(*E*) could be estimated from all *e_ij _*values, where *E *= {*e_ij _*| *i *= 1,..., *n*-1, *j *= *i*+1,..., *n*, and *n *= total number of genes of interest} and the probability of having the similarity less than or equal to *e_ij_*, Pr(*E*≤*e_ij_*) could be obtained from *F*(*e_ij_*). With this method, a large *e_ij _*value from highly similar pair of genes has a high probability - close to one. The probabilities for *t_ij_*, Pr(*T*≤*t_ij_*) and *g_ij_*, Pr(*G*≤*g_ij_*) could be obtained similarly to *e_ij_*. Next, the joint probability was calculated by simply multiplying three probabilities in accord with the independence assumption, i.e. Pr(*E*≤*e_ij_*, *T*≤*t_ij_*, *G*≤*g_ij_*) = Pr(*E*≤*e_ij_*)·Pr(*T*≤*t_ij_*)·Pr(*G*≤*g_ij_*). This is then used as a composite similarity measure.

### Clustering method

Affinity propagation is a clustering algorithm using the concept of message-passing [27]. It was modeled using a factor graph [41] with two types of messages between data points (in this study the points were genes) that were derived from the max-sum algorithm in the factor graph. First consider all data points as potential cluster centers and then find high quality cluster centers and their members by updating messages. The detailed description of the method can be found in [27,42]. Since affinity propagation takes non-positive real-valued similarities as input, the combined similarity was converted into *S_ij _*= Pr(*E*≤*e_ij_*, *T*≤*t_ij_*, *G*≤*g_ij_*)-1, *S_ij_*∈[-1,0]. The only parameter affecting the number of clusters is the "preference" which represents how "preferable" each data point is as a cluster center. We used a globally shared preference for all genes and found a proper value for the comparison study in order to produce the same number of modules as other methods, so that the comparisons could be made. We used authors' MATLAB implementations of affinity propagation http://www.psi.toronto.edu/affinitypropagation.

### Gene ontology enrichment analysis

Gene ontology data were downloaded from GO consortium (http://www.geneontology.org, OBO v1.2 format) and MIPS FTP site (ftp://ftpmips.gsf.de/catalogue/annotation_data, FunCat v2.1 last modified on 5/25/2008). Yeast gene association with gene ontology and gene information data were obtained from the NCBI repository on 2/17/2010 and 9/5/2010, respectively. *P*-value of enriched annotation term was obtained by one-sided Fisher exact test without multiple testing corrections. The number of enriched annotation terms in at least one module at a given significance level (i.e. *p *< 1 × 10^-4^) and the number of modules enriched with at least one annotation term at the level were counted and used for computing sensitivity and specificity, respectively. In Additional File 1, these numbers were expressed as functions of *p*-values (*p *= [10^-1^, 10^-2^,..., 10^-20^]).

### Coherence and depth of enriched gene ontology terms

For each method used in the comparison study, enriched GO BP terms in *i*th module having uncorrected *p*-values less than 1 × 10^-5 ^were chosen. All pairwise semantic similarities between the terms, *g_BP,jk _*(*j *= 1,..., *m_i_*-1 and *k *= *j*+1,... *m_i_*, where *m_i _*= the number of chosen terms from *i*th module) were calculated by Wang's approach [25] and the depth of each term, *d_BP,j _*(*j *= 1,... *m_i_*) was obtained using the shortest path to the root term (GO:0008150 biological process) in GO hierarchy. We could obtain representative coherency and depth of enriched GO BP terms for the *i*th module by *G_BP,i _*= 2/*m_i_*(*m_i_*-1)·∑_*j*_∑_*k *_*g_BP,jk _*and *D_BP,i _*= 1/*m_i_*·∑_*j *_*d_BP,j_*, respectively (*i *= 1,..., total number of modules identified by the method). Mean values of *G_BP _*and *D_BP _*over all modules were defined as average coherency and depth of enriched GO terms and shown in Additional File 2.

### Comparison of the modules identified by different methods

For MATISSE and ICMg, due to their probabilistic nature, it is not possible to make a unique assignment of genes into modules. Instead, the most likely assignment can be identified using consensus matrix of which element at *i*th row and *j*th column represents how many times gene *i *and gene *j *are grouped into the same module among 20 runs. Using hierarchical clustering of the consensus matrix, the assignment of genes into 24 modules was obtained and used for the comparison between ICMg module and SSIM modules (see text and Additional File 3).

### Extension of a module

A module identified by SSIM can be extended according to the following procedure.

Step 1. find neighbor genes which have direct interactions with more than one gene in the module, but are not in the selected gene set. Sort them in descending order based on the number of interactions with genes in the module

Step 2. for the top scoring neighbor gene, GO semantic similarities between the gene and all genes in the module were calculated

Step 3. if the mean value of the similarities computed in Step 2 is larger than the mean semantic similarity among genes in the module, the neighbor gene is added to the module

Step 4. go back to Step 2 until the genes are all classified

This procedure imposes a priority to the neighbor genes having many connections with genes in the module (Step 1) and minimizes the involvement of irrelevant neighbor genes by increasing average semantic similarity of the module at every addition of a relevant neighbor gene (Step 3). Although we only used semantic similarity between the neighbor gene and genes in the module due to the limitation of expression profiles, expression similarity can be also added to Step 2 and 3 when gene expression of neighbors are available.

## Competing interests

The authors declare that they have no competing interests.

## Authors' contributions

JHC, KW and DJG co-conceived the study. JHC developed the method and performed data analyses. JHC and KW wrote the initial manuscript, DJG edited the manuscript. All of the authors read and approved the final manuscript.

## Supplementary Material

Additional file 1 **Additional enrichment results**. The number of enriched modules, number of annotation terms and F-measures (GO, GO BP and MIPS FunCat) were shown as functions of *p*-value. Results for MATISSE and ICMg were obtained using the mean and standard deviation values of 20 runs. Click here for file

Additional file 2 **Coherency and depth of significantly enriched GO BP terms**. Average expression coherency and depth of significantly enriched GO BP terms in the modules identified by different methods were calculated as described in the method section. Large coherency and depth values mean that GO BP terms enriched in the same module are semantically similar and associated with specific functions, respectively. For MATISSE and ICMg, mean and standard deviation over 20 runs were taken. Click here for file

Additional file 3 **Summary of the modules identified by three different methods**. The assignments of 1990 genes in MATISSE data into 24 modules identified by SSIM, MATISSE and ICMg were summarized in Table S1 (see methods). GO BP enrichment results of the modules obtained from SSIM, MATISSE and ICMg were also shown in Table S2, S3 and S4, respectively. Click here for file

Additional file 4 **Results of SSIM method over a wide range of preference values**. The number of modules and average expression, topological and semantic similarities of the modules were expressed as functions of preference. Click here for file

Additional file 5 An example of the extension of a module Click here for file

Additional file 6 An application of SSIM to prion network datasets Click here for file

## References

[B1] Hood L Heath JR Phelps ME Lin B Systems biology and new technologies enable predictive and preventative medicine Science 2004 306 640 643 10.1126/science.1104635 15499008

[B2] Wang K Lee I Carlson G Hood L Galas D Systems biology and the discovery of diagnostic biomarkers Dis Markers 2010 28 199 207 20534905 10.3233/DMA-2010-0697 PMC3021550

[B3] Hartwell LH Hopfield JJ Leibler S Murray AW From molecular to modular cell biology Nature 1999 402 C47 52 10.1038/35011540 10591225

[B4] Eisen MB Spellman PT Brown PO Botstein D Cluster analysis and display of genome-wide expression patterns Proc Natl Acad Sci USA 1998 95 14863 14868 10.1073/pnas.95.25.14863 9843981 PMC24541

[B5] Spellman PT Sherlock G Zhang MQ Iyer VR Anders K Eisen MB Brown PO Botstein D Futcher B Comprehensive identification of cell cycle-regulated genes of the yeast Saccharomyces cerevisiae by microarray hybridization Mol Biol Cell 1998 9 3273 3297 9843569 10.1091/mbc.9.12.3273 PMC25624

[B6] Alon U Barkai N Notterman DA Gish K Ybarra S Mack D Levine AJ Broad patterns of gene expression revealed by clustering analysis of tumor and normal colon tissues probed by oligonucleotide arrays Proc Natl Acad Sci USA 1999 96 6745 6750 10.1073/pnas.96.12.6745 10359783 PMC21986

[B7] Iyer VR Eisen MB Ross DT Schuler G Moore T Lee JC Trent JM Staudt LM Hudson J Jr Boguski MS The transcriptional program in the response of human fibroblasts to serum Science 1999 283 83 87 10.1126/science.283.5398.83 9872747

[B8] Sharan R Maron-Katz A Shamir R CLICK and EXPANDER: a system for clustering and visualizing gene expression data Bioinformatics 2003 19 1787 1799 10.1093/bioinformatics/btg232 14512350

[B9] Ge H Liu Z Church GM Vidal M Correlation between transcriptome and interactome mapping data from Saccharomyces cerevisiae Nat Genet 2001 29 482 486 10.1038/ng776 11694880

[B10] Ideker T Ozier O Schwikowski B Siegel AF Discovering regulatory and signalling circuits in molecular interaction networks Bioinformatics 2002 18 Suppl 1 S233 240 10.1093/bioinformatics/18.suppl_1.S233 12169552

[B11] Shiga M Takigawa I Mamitsuka H Annotating gene function by combining expression data with a modular gene network Bioinformatics 2007 23 i468 478 10.1093/bioinformatics/btm173 17646332

[B12] Ulitsky I Shamir R Identification of functional modules using network topology and high-throughput data BMC Syst Biol 2007 1 8 10.1186/1752-0509-1-8 17408515 PMC1839897

[B13] Ulitsky I Shamir R Identifying functional modules using expression profiles and confidence-scored protein interactions Bioinformatics 2009 25 1158 1164 10.1093/bioinformatics/btp118 19297352

[B14] Parkkinen JA Kaski S Searching for functional gene modules with interaction component models BMC Syst Biol 2010 4 4 10.1186/1752-0509-4-4 20100324 PMC2823677

[B15] Gu J Chen Y Li S Li Y Identification of responsive gene modules by network-based gene clustering and extending: application to inflammation and angiogenesis BMC Syst Biol 2010 4 47 10.1186/1752-0509-4-47 20406493 PMC2873318

[B16] Heyer LJ Kruglyak S Yooseph S Exploring expression data: identification and analysis of coexpressed genes Genome Res 1999 9 1106 1115 10.1101/gr.9.11.1106 10568750 PMC310826

[B17] Wang Z Zhang J In search of the biological significance of modular structures in protein networks PLoS Comput Biol 2007 3 e107 10.1371/journal.pcbi.0030107 17542644 PMC1885274

[B18] Jiang J Conrath D Semantic similarity based on corpus statistics and lexical taxonomy Proceedings of International Conference Research on Computational Linguistics (ROCLING X); Taiwan 1997

[B19] Lin D An information-theoretic definition of similarity Proceedings of the Fifteenth International Conference on Machine Learning; USA 1998

[B20] Resnik P Semantic similarity in a taxonomy: an information-based measure and its application to problems of ambiguity in natural language J Artificial Intelligence Res 1999 11 95 130

[B21] Lee PH Lee D Modularized learning of genetic interaction networks from biological annotations and mRNA expression data Bioinformatics 2005 21 2739 2747 10.1093/bioinformatics/bti406 15797909

[B22] Wu X Zhu L Guo J Zhang DY Lin K Prediction of yeast protein-protein interaction network: insights from the Gene Ontology and annotations Nucleic Acids Res 2006 34 2137 2150 10.1093/nar/gkl219 16641319 PMC1449908

[B23] Guo X Liu R Shriver CD Hu H Liebman MN Assessing semantic similarity measures for the characterization of human regulatory pathways Bioinformatics 2006 22 967 973 10.1093/bioinformatics/btl042 16492685

[B24] Cho YR Shi L Ramanathan M Zhang A A probabilistic framework to predict protein function from interaction data integrated with semantic knowledge BMC Bioinformatics 2008 9 382 10.1186/1471-2105-9-382 18801191 PMC2570367

[B25] Wang JZ Du Z Payattakool R Yu PS Chen CF A new method to measure the semantic similarity of GO terms Bioinformatics 2007 23 1274 1281 10.1093/bioinformatics/btm087 17344234

[B26] Ashburner M Ball CA Blake JA Botstein D Butler H Cherry JM Davis AP Dolinski K Dwight SS Eppig JT Gene ontology: tool for the unification of biology. The Gene Ontology Consortium Nat Genet 2000 25 25 29 10.1038/75556 10802651 PMC3037419

[B27] Frey BJ Dueck D Clustering by passing messages between data points Science 2007 315 972 976 10.1126/science.1136800 17218491

[B28] Ruepp A Zollner A Maier D Albermann K Hani J Mokrejs M Tetko I Guldener U Mannhaupt G Munsterkotter M Mewes HW The FunCat, a functional annotation scheme for systematic classification of proteins from whole genomes Nucleic Acids Res 2004 32 5539 5545 10.1093/nar/gkh894 15486203 PMC524302

[B29] Barabasi AL Oltvai ZN Network biology: understanding the cell's functional organization Nat Rev Genet 2004 5 101 113 10.1038/nrg1272 14735121

[B30] Lee TI Rinaldi NJ Robert F Odom DT Bar-Joseph Z Gerber GK Hannett NM Harbison CT Thompson CM Simon I Transcriptional regulatory networks in Saccharomyces cerevisiae Science 2002 298 799 804 10.1126/science.1075090 12399584

[B31] Segal E Shapira M Regev A Pe'er D Botstein D Koller D Friedman N Module networks: identifying regulatory modules and their condition-specific regulators from gene expression data Nat Genet 2003 34 166 176 10.1038/ng1165 12740579

[B32] Stuart JM Segal E Koller D Kim SK A gene-coexpression network for global discovery of conserved genetic modules Science 2003 302 249 255 10.1126/science.1087447 12934013

[B33] Lee SI Dudley AM Drubin D Silver PA Krogan NJ Pe'er D Koller D Learning a prior on regulatory potential from eQTL data PLoS Genet 2009 5 e1000358 10.1371/journal.pgen.1000358 19180192 PMC2627940

[B34] Hwang D Lee IY Yoo H Gehlenborg N Cho JH Petritis B Baxter D Pitstick R Young R Spicer D A systems approach to prion disease Mol Syst Biol 2009 5 252 19308092 10.1038/msb.2009.10 PMC2671916

[B35] Wang J Zhou X Zhu J Zhou C Guo Z Revealing and avoiding bias in semantic similarity scores for protein pairs BMC Bioinformatics 2010 11 290 10.1186/1471-2105-11-290 20509916 PMC2903568

[B36] Benabderrahmane S Smail-Tabbone M Poch O Napoli A Devignes MD IntelliGO: a new vector-based semantic similarity measure including annotation origin BMC Bioinformatics 2010 11 588 10.1186/1471-2105-11-588 21122125 PMC3098105

[B37] Jain S Bader GD An improved method for scoring protein-protein interactions using semantic similarity within the gene ontology BMC Bioinformatics 2010 11 562 10.1186/1471-2105-11-562 21078182 PMC2998529

[B38] O'Rourke SM Herskowitz I Unique and redundant roles for HOG MAPK pathway components as revealed by whole-genome expression analysis Mol Biol Cell 2004 15 532 542 14595107 10.1091/mbc.E03-07-0521 PMC329229

[B39] Ravasz E Somera AL Mongru DA Oltvai ZN Barabasi AL Hierarchical organization of modularity in metabolic networks Science 2002 297 1551 1555 10.1126/science.1073374 12202830

[B40] Pesquita C Faria D Falcao AO Lord P Couto FM Semantic similarity in biomedical ontologies PLoS Comput Biol 2009 5 e1000443 10.1371/journal.pcbi.1000443 19649320 PMC2712090

[B41] Kschischang FR Frey BJ Loeliger H-A Factor graphs and the sum-product algorithm IEEE Trans Inf Theory 2001 47 498 519 10.1109/18.910572

[B42] Dueck D Affinity propagation: clustering data by passing messages PhD thesis 2009 University of Toronto, Department of electrical & computer engineering

