# Supplementary material for: An integrative approach to inferring biologically meaningful gene modules
Source: BMC Syst Biol. 2011 Jul 26;5:117. doi: 10.1186/1752-0509-5-117 (PMC3156758; doi:10.1186/1752-0509-5-117)
Supplement: Additional file 1 — Additional enrichment results. The number of enriched modules, number of annotation terms and F-measures (GO, GO BP and MIPS FunCat) were shown as functions of p-value. Results for MATISSE and ICMg were obtained using the mean and standard deviation values of 20 runs. [file 1752-0509-5-117-S1.PDF]

Additional File 1. The performance of each method is evaluated by enrichment study using three annotations, all GO terms (upper), GO BP terms (middle) and MIPS FunCat terms (lower panel) and expressed using the number of enriched modules (left), number of enriched annotation terms (middle) and *F*-measure (right) as functions of various p-values. See methods for the details. Results for MATISSE and ICMg were obtained using the mean and standard deviation values of 20 runs.

MATISSE data

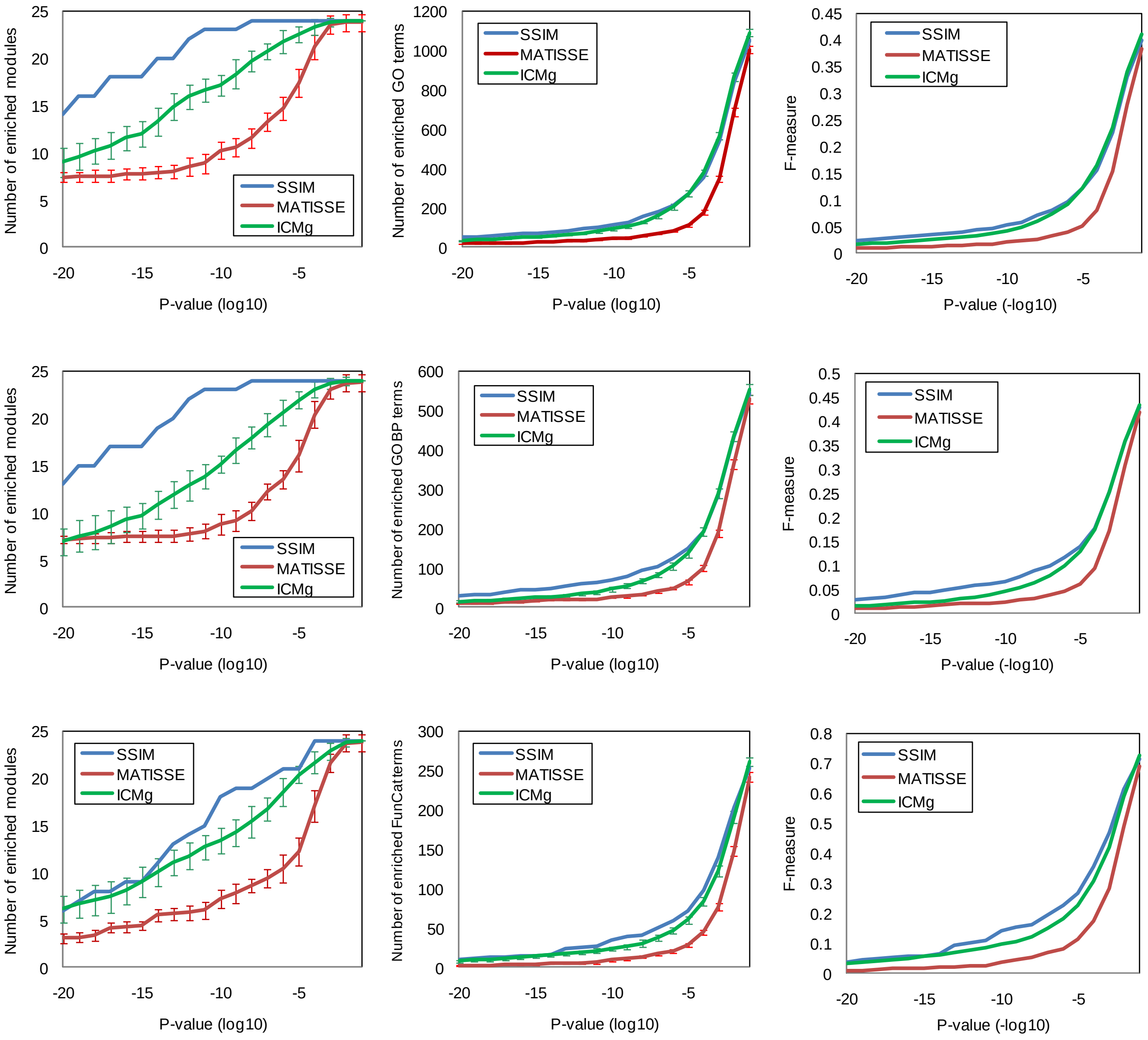

ICMg data

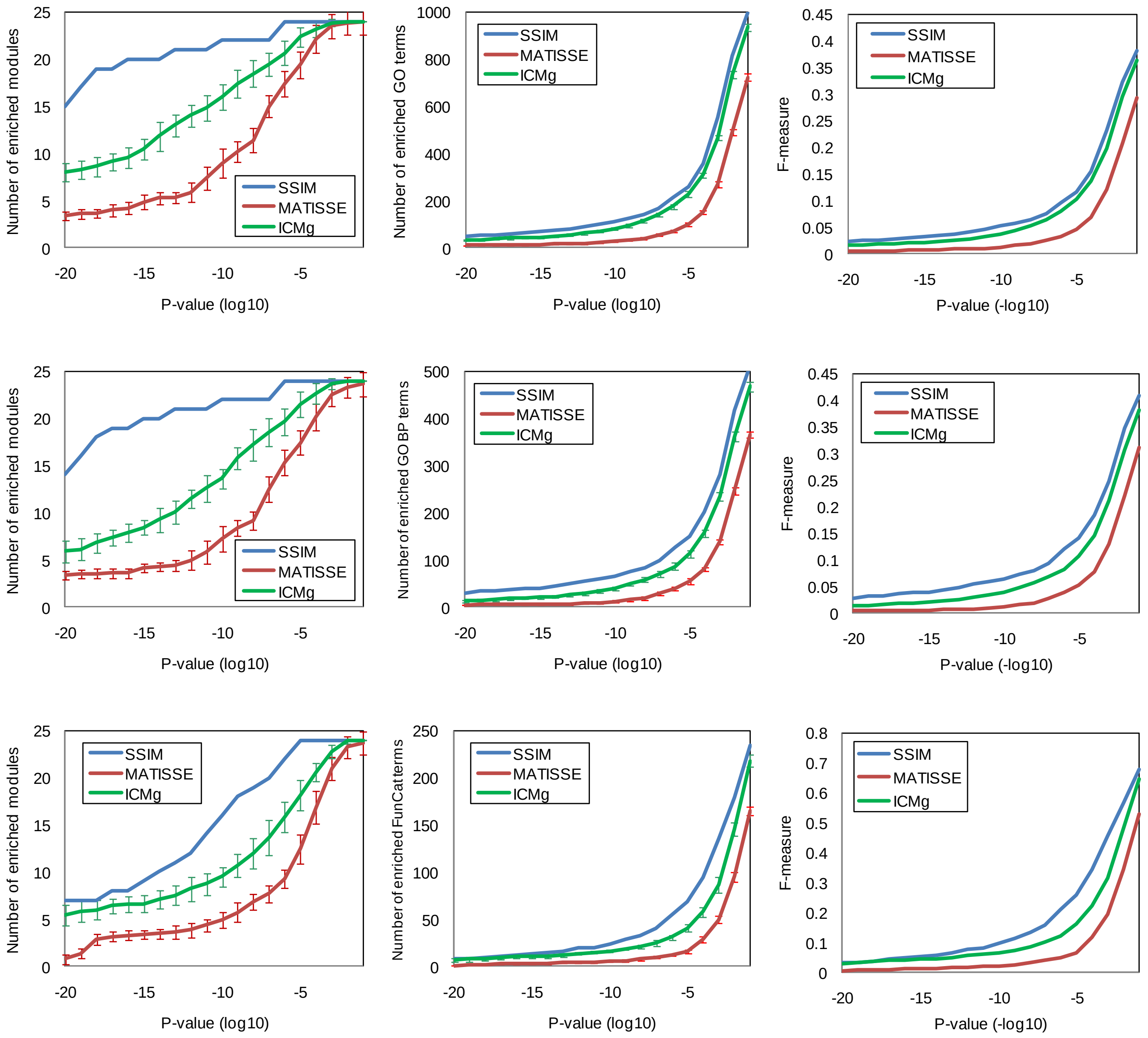

Number of enriched modules
P-value (log10)
SSIM
MATISSE
ICMg
Number of enriched GO terms
P-value (log10)
SSIM
MATISSE
ICMg
F-measure
P-value (-log10)
SSIM
MATISSE
ICMg
Number of enriched modules
P-value (log10)
SSIM
MATISSE
ICMg
Number of enriched GO BP terms
P-value (log10)
SSIM
MATISSE
ICMg
F-measure
P-value (-log10)
SSIM
MATISSE
ICMg
Number of enriched modules
P-value (log10)
SSIM
MATISSE
ICMg
Number of enriched FunCat terms
P-value (log10)
SSIM
MATISSE
ICMg
F-measure
P-value (-log10)
SSIM
MATISSE
ICMg
