# Supplementary material for: An integrative approach to inferring biologically meaningful gene modules
Source: BMC Syst Biol. 2011 Jul 26;5:117. doi: 10.1186/1752-0509-5-117 (PMC3156758; doi:10.1186/1752-0509-5-117)

Additional File 2. Average coherency and depth of significantly enriched GO BP terms (at $p<1.0\times10^{-4}$) in the modules identified by methods are described (see methods). Large coherency and depth values mean that GO BP terms enriched in the same module are semantically similar and associated with specific functions, respectively. For MATISSE and ICMg, mean and standard deviation over 20 runs were taken.

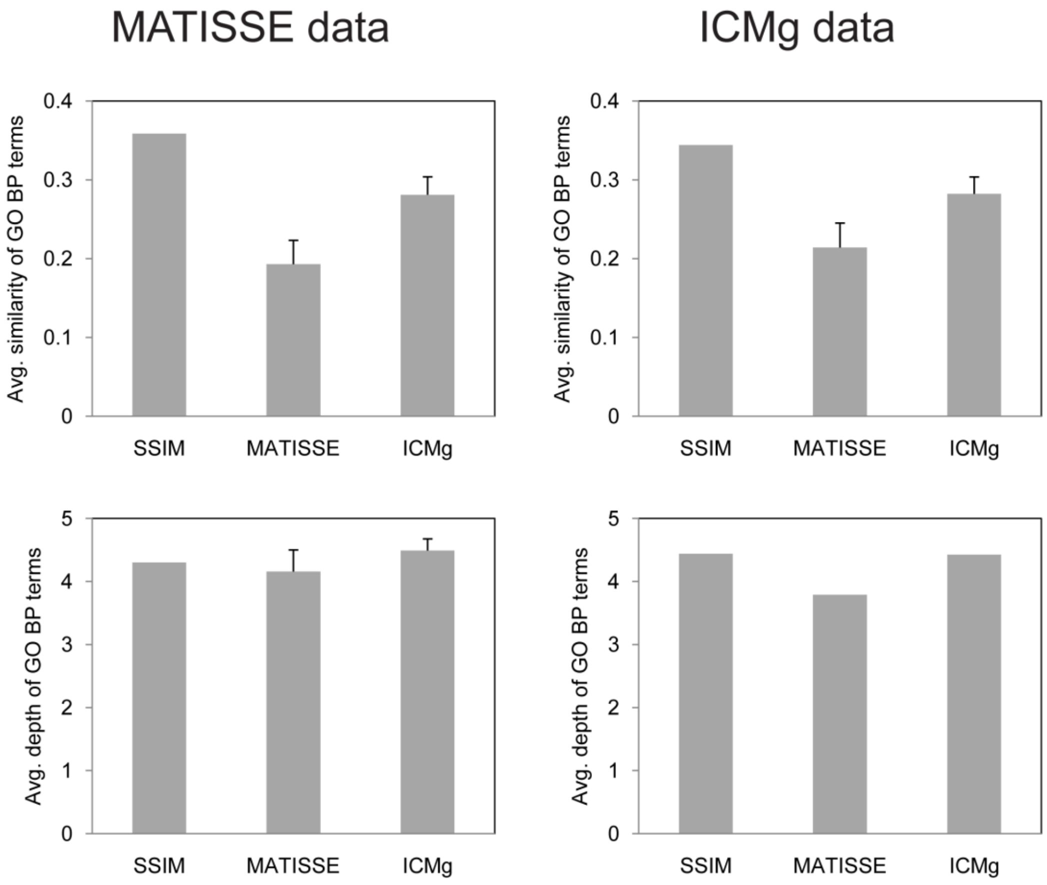

Supplement: Additional file 2 — Coherency and depth of significantly enriched GO BP terms. Average expression coherency and depth of significantly enriched GO BP terms in the modules identified by different methods were calculated as described in the method section. Large coherency and depth values mean that GO BP terms enriched in the same module are semantically similar and associated with specific functions, respectively. For MATISSE and ICMg, mean and standard deviation over 20 runs were taken. [file 1752-0509-5-117-S2.PDF]
