# Supplementary material for: An integrative approach to inferring biologically meaningful gene modules
Source: BMC Syst Biol. 2011 Jul 26;5:117. doi: 10.1186/1752-0509-5-117 (PMC3156758; doi:10.1186/1752-0509-5-117)

Additional File 4. According to Frey and Dueck [29], the number of modules (i.e. clusters) unchanged over some preference values can be considered as sub-optimum. We applied SSIM method to MATISSE data with a wide range of preference values (-15~-0.5, 0.1 increment) and obtained the number of modules, average expression, topological and semantic similarities (blue, red and green line, respectively) as functions of preference. At 12, 18 and 37 modules, the number of modules as well as three similarities showed stable behaviors over some preference values.

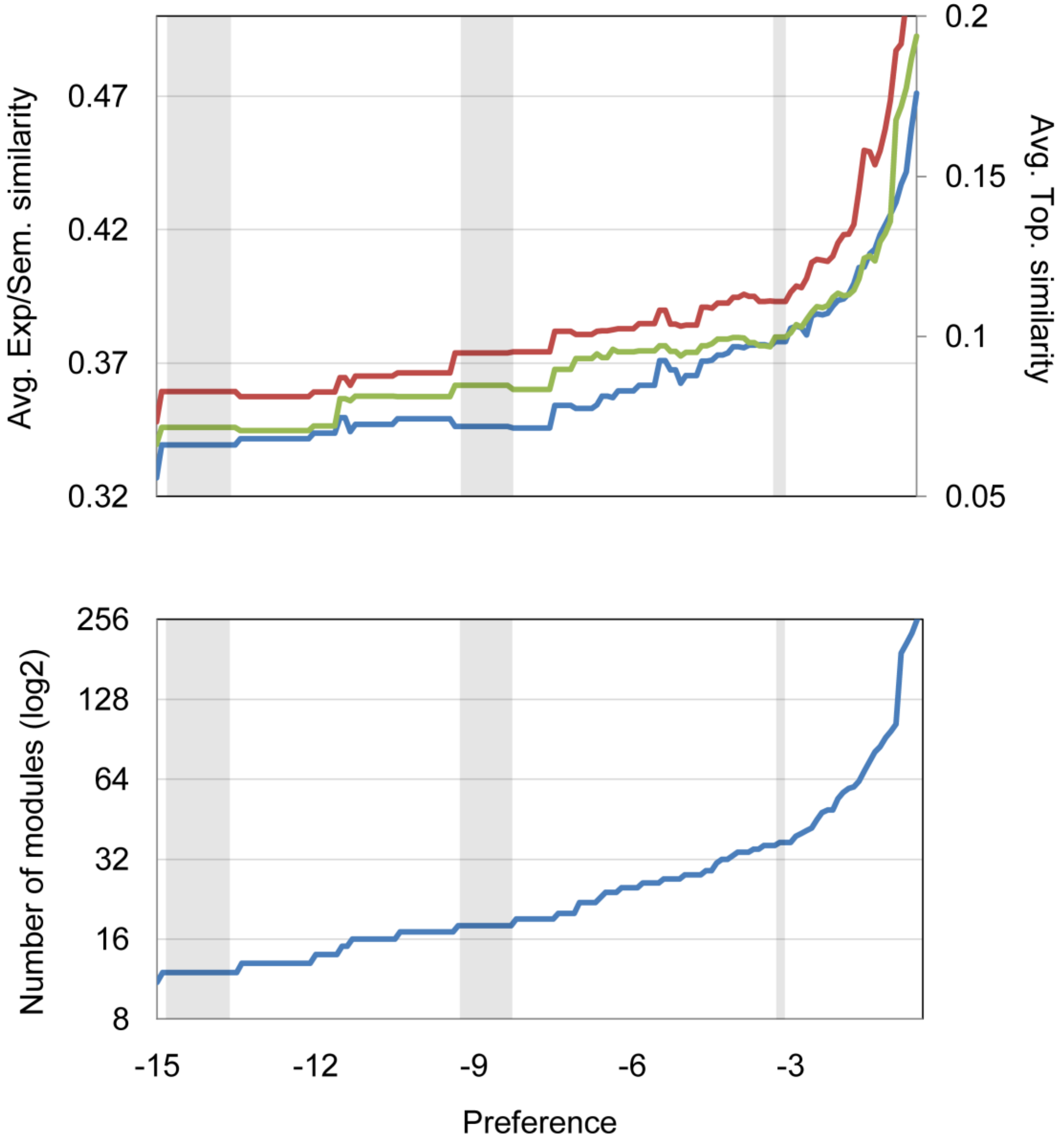

Supplement: Additional file 4 — Results of SSIM method over a wide range of preference values. The number of modules and average expression, topological and semantic similarities of the modules were expressed as functions of preference. [file 1752-0509-5-117-S4.PDF]
