# Supplementary material for: An integrative approach to inferring biologically meaningful gene modules
Source: BMC Syst Biol. 2011 Jul 26;5:117. doi: 10.1186/1752-0509-5-117 (PMC3156758; doi:10.1186/1752-0509-5-117)
Supplement: Additional file 5 — An example of the extension of a module [file 1752-0509-5-117-S5.PDF]

Additional File 5. A network for the module representing cell cycle related functions were depicted in the left panel of the figure. Originally, the module had 72 genes (white nodes) with only 47 interactions (black lines). Through the extension procedure, 55 genes (red nodes) were added and produced additional 264 interactions (red lines) as shown on the right. GO enrichment results before and after the extension were also shown under the module networks.

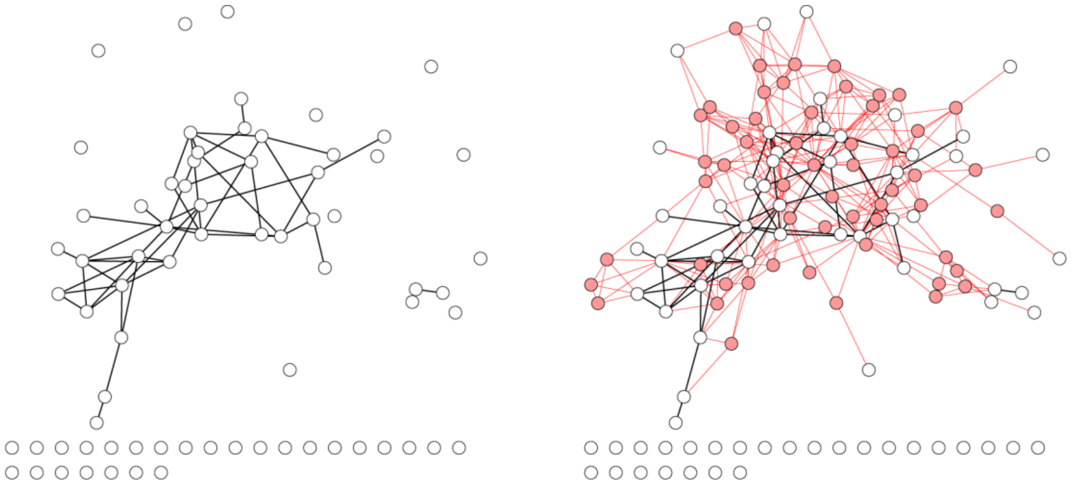

GO ID	GO Term	P-value
GO:0051301	cell division	3.72E-23
GO:0007049	cell cycle	1.13E-22
GO:0007067	mitosis	1.57E-18
GO:0000278	mitotic cell cycle	1.26E-11
GO:0050790	regulation of catalytic activity	2.28E-11

Before extension

GO ID	GO Term	P-value
GO:0007049	cell cycle	8.07E-64
GO:0007067	mitosis	6.34E-59
GO:0051301	cell division	3.90E-56
GO:0000278	mitotic cell cycle	7.05E-54
GO:0000279	M phase	1.67E-52

After extension
