# Supplementary material for: An integrative approach to inferring biologically meaningful gene modules
Source: BMC Syst Biol. 2011 Jul 26;5:117. doi: 10.1186/1752-0509-5-117 (PMC3156758; doi:10.1186/1752-0509-5-117)
Supplement: Additional file 6 — An application of SSIM to prion network datasets [file 1752-0509-5-117-S6.PDF]

Additional File 6. Application of SSIM to prion network dataset

SSIM was applied to the 648 genes in four prion subnetworks (microglial activation, PrPsc accumulation, neuronal cell death and synaptic degeneration). In order to compute the integrated similarity, temporal fold-changes of the genes [1, 2], interaction data obtained from HPRD, BioGRID, Reactome, MINT and KEGG database and GO annotation of mouse genome were used. Given n (= 648) genes and the integrated similarity matrix $\mathbf{S}\in R^{n\times n}$, the following procedure was used in order to determine the proper number of modules.

Step 1. Initialize the count of iteration $k = 0$, membership matrix $\mathbf{M}\in R^{n\times n}$ in which each element $M_{ij} = 0$ for $i, j = 1,\ldots,n$ and the preference value, r (in this study -20 was used)

Step 2. Affinity propagation was run using the current preference value r and similarity matrix **S**

Step 3. For i and j ($i, j = 1,\ldots,n$), membership value between gene i and gene j was computed as following,

$M_{ij} = M_{ij}+1$, if two genes were assigned to the same module

$M_{ij} = M_{ij}$, otherwise

Step 4. Increase the preference value, $r = r+0.1$

Step 5. Count the number of iterations, $k = k+1$

Step 5. Iterate step 2~4 until r reaches the pre-determined value (-1 for this dataset)

Step 6. Divide all the elements in **M** by k in order to make **M** be bounded between 0 and 1

M_{ij} value close to 1 means that gene i and gene j tend to be grouped together and M_{ij} value close to 0 means that two genes are not likely to be grouped into the same module, with respect to various numbers of modules. Therefore, the membership matrix could reflect the underlying groups of genes. Using hierarchical clustering of the matrix, 16 modules for the prion network dataset were identified as shown in Figure S1. The number and proportion of genes in the modules overlapped with four subnetworks are summarized in Table 1 in main text and GO enrichment result of the modules is shown in Table S1. Several modules identified by SSIM effectively reflect a part of subnetwork. For example, module 1 and 3 are composed of genes related to PrP accumulation subnetwork, module 2, 7, 11, 13 and 14 correspond to a large portion of microglial activation subnetwork and so forth. Figure S2, 3, 4 and 5, originated from [1], describe the genes in subnetworks which correspond to SSIM modules.

Table S1. GO BP enrichment result of 16 modules identified from SSIM

Module	GO ID	GO BP Term	P-value
1	GO:0006508	proteolysis	6.73E-13
1	GO:0006629	lipid metabolic process	1.91E-07
1	GO:0008203	cholesterol metabolic process	4.72E-06
1	GO:0006694	steroid biosynthetic process	4.99E-06
2	GO:0007155	cell adhesion	9.85E-21
2	GO:0007229	integrin-mediated signaling pathway	1.45E-09
2	GO:0050798	activated T cell proliferation	1.21E-08
2	GO:0007159	leukocyte adhesion	7.59E-08
2	GO:0030593	neutrophil chemotaxis	1.93E-07
2	GO:0033627	cell adhesion mediated by integrin	5.17E-07
2	GO:0045123	cellular extravasation	3.10E-06
2	GO:0007160	cell-matrix adhesion	3.36E-06
2	GO:0016337	cell-cell adhesion	8.86E-06
3	GO:0016126	sterol biosynthetic process	4.22E-22
3	GO:0006694	steroid biosynthetic process	2.68E-19
3	GO:0008610	lipid biosynthetic process	3.21E-17
3	GO:0006695	cholesterol biosynthetic process	4.07E-16
3	GO:0008299	isoprenoid biosynthetic process	2.01E-14
3	GO:0016310	phosphorylation	9.15E-06
4	GO:0007275	multicellular organismal development	1.45E-08
4	GO:0030154	cell differentiation	7.77E-08
4	GO:0001525	angiogenesis	2.26E-07
4	GO:0006915	apoptosis	7.79E-06
5	GO:0030036	actin cytoskeleton organization	3.77E-08
5	GO:0006897	endocytosis	4.38E-07
5	GO:0001525	angiogenesis	6.13E-07
5	GO:0007040	lysosome organization	1.49E-06
5	GO:0030032	lamellipodium assembly	1.49E-06
5	GO:0006909	phagocytosis	3.86E-06
5	GO:0060055	angiogenesis involved in wound healing	5.24E-06
5	GO:0007264	small GTPase mediated signal transduction	5.28E-06
6	GO:0045449	regulation of cellular transcription	1.49E-20
6	GO:0006350	cellular transcription	6.01E-20
6	GO:0006355	regulation of cellular transcription, DNA-dependent	4.17E-13
6	GO:0045944	positive regulation of transcription from RNA polymerase II promoter	6.85E-13
6	GO:0035162	embryonic hemopoiesis	1.46E-07
6	GO:0016573	histone acetylation	2.68E-07
6	GO:0001892	embryonic placenta development	1.61E-06
6	GO:0000122	negative regulation of transcription from RNA polymerase II promoter	8.18E-06
7	GO:0006468	protein amino acid phosphorylation	4.22E-14
7	GO:0007242	intracellular signaling cascade	2.85E-11
7	GO:0018108	peptidyl-tyrosine phosphorylation	3.37E-10
7	GO:0050853	B cell receptor signaling pathway	5.08E-10
7	GO:0046641	positive regulation of alpha-beta T cell proliferation	1.35E-07
7	GO:0046777	protein amino acid autophosphorylation	2.54E-07
7	GO:0043366	beta selection	9.09E-07
7	GO:0006654	phosphatidic acid biosynthetic process	2.73E-06
7	GO:0050732	negative regulation of peptidyl-tyrosine phosphorylation	5.45E-06
7	GO:0045577	regulation of B cell differentiation	9.08E-06
7	GO:0045588	positive regulation of gamma-delta T cell differentiation	9.08E-06
8	GO:0006810	transport	1.11E-15
8	GO:0006811	ion transport	9.23E-09
8	GO:0016079	synaptic vesicle exocytosis	1.23E-07
8	GO:0055085	transmembrane transport	5.69E-07
9	GO:0006915	apoptosis	3.96E-47
9	GO:0042981	regulation of apoptosis	4.74E-37
9	GO:0006917	induction of apoptosis	2.16E-16
9	GO:0043066	negative regulation of apoptosis	1.31E-14
9	GO:0043065	positive regulation of apoptosis	5.54E-13
9	GO:0001836	release of cytochrome c from mitochondria	4.14E-11
9	GO:0006508	proteolysis	5.05E-11
9	GO:0001782	B cell homeostasis	5.86E-11
9	GO:0007507	heart development	1.20E-10
9	GO:0043029	T cell homeostasis	6.31E-09
9	GO:0051402	neuron apoptosis	1.14E-08
9	GO:0070059	apoptosis in response to endoplasmic reticulum stress	1.66E-08

9	GO:0048538	thymus development	9.29E-08
9	GO:0001776	leukocyte homeostasis	1.44E-07
9	GO:0002262	myeloid cell homeostasis	2.31E-07
9	GO:0006309	DNA fragmentation involved in apoptosis	6.77E-07
9	GO:0042542	response to hydrogen peroxide	6.77E-07
9	GO:0008283	cell proliferation	7.82E-07
9	GO:0051726	regulation of cell cycle	1.63E-06
9	GO:0043524	negative regulation of neuron apoptosis	1.76E-06
9	GO:0006979	response to oxidative stress	2.05E-06
9	GO:0016337	cell-cell adhesion	2.05E-06
9	GO:0033077	T cell differentiation in the thymus	3.32E-06
9	GO:0009411	response to UV	8.19E-06
10	GO:0045103	intermediate filament-based process	4.00E-06
11	GO:0006955	immune response	3.99E-12
11	GO:0006935	chemotaxis	1.54E-11
11	GO:0006954	inflammatory response	2.40E-08
11	GO:0007165	signal transduction	6.26E-08
12	GO:0006915	apoptosis	2.72E-17
12	GO:0042981	regulation of apoptosis	3.50E-14
12	GO:0006917	induction of apoptosis	4.45E-11
12	GO:0008624	induction of apoptosis by extracellular signals	5.01E-09
12	GO:0006468	protein amino acid phosphorylation	6.28E-08
12	GO:0043123	positive regulation of I-kappaB kinase/NF-kappaB cascade	1.14E-07
12	GO:0007254	JNK cascade	1.86E-06
12	GO:0006916	anti-apoptosis	2.83E-06
12	GO:0019987	negative regulation of anti-apoptosis	6.06E-06
12	GO:0006919	activation of caspase activity	7.05E-06
13	GO:0006955	immune response	2.09E-33
13	GO:0045087	innate immune response	4.82E-24
13	GO:0006958	complement activation, classical pathway	8.10E-18
13	GO:0050766	positive regulation of phagocytosis	2.91E-11
13	GO:0006911	phagocytosis, engulfment	2.40E-09
13	GO:0042590	antigen processing and presentation of exogenous peptide antigen via MHC class I	6.42E-09
13	GO:0006956	complement activation	1.60E-08
13	GO:0006953	acute-phase response	1.85E-08
13	GO:0001798	positive regulation of type IIa hypersensitivity	8.95E-08
13	GO:0019882	antigen processing and presentation	2.12E-07
13	GO:0006910	phagocytosis, recognition	2.63E-07
13	GO:0045576	mast cell activation	2.63E-07
13	GO:0002474	antigen processing and presentation of peptide antigen via MHC class I	7.23E-07
13	GO:0019886	antigen processing and presentation of exogenous peptide antigen via MHC class II	8.90E-07
13	GO:0016064	immunoglobulin mediated immune response	2.43E-06
13	GO:0001805	positive regulation of type III hypersensitivity	4.23E-06
13	GO:0014909	smooth muscle cell migration	4.23E-06
13	GO:0001788	antibody-dependent cellular cytotoxicity	8.45E-06
14	GO:0045944	positive regulation of transcription from RNA polymerase II promoter	4.40E-17
14	GO:0045449	regulation of cellular transcription	2.55E-15
14	GO:0006350	cellular transcription	1.17E-13
14	GO:0006355	regulation of cellular transcription, DNA-dependent	1.98E-13
14	GO:0045893	positive regulation of cellular transcription, DNA-dependent	1.48E-12
14	GO:0006954	inflammatory response	1.61E-10
14	GO:0007179	transforming growth factor beta receptor signaling pathway	1.70E-10
14	GO:0051092	positive regulation of NF-kappaB transcription factor activity	9.06E-10
14	GO:0010468	regulation of gene expression	2.23E-09
14	GO:0042127	regulation of cell proliferation	1.73E-08
14	GO:0009617	response to bacterium	2.40E-08
14	GO:0050793	regulation of developmental process	5.88E-08
14	GO:0032760	positive regulation of tumor necrosis factor production	1.26E-07
14	GO:0032755	positive regulation of interleukin-6 production	1.49E-07
14	GO:0008285	negative regulation of cell proliferation	2.92E-07
14	GO:0043011	myeloid dendritic cell differentiation	3.28E-07
14	GO:0001501	skeletal system development	3.56E-07
14	GO:0032494	response to peptidoglycan	4.92E-07
14	GO:0048147	negative regulation of fibroblast proliferation	4.92E-07
14	GO:0060021	palate development	5.79E-07
14	GO:0006917	induction of apoptosis	6.12E-07
14	GO:0045941	positive regulation of cellular transcription	8.50E-07
14	GO:0019221	cytokine-mediated signaling pathway	9.89E-07
14	GO:0045087	innate immune response	1.05E-06
14	GO:0006955	immune response	1.20E-06

14	GO:0030099	myeloid cell differentiation	1.28E-06
14	GO:0031663	lipopolysaccharide-mediated signaling pathway	1.28E-06
14	GO:0010552	positive regulation of gene-specific transcription from RNA polymerase II promoter	1.73E-06
14	GO:0032496	response to lipopolysaccharide	2.60E-06
14	GO:0051726	regulation of cell cycle	2.60E-06
14	GO:0007219	Notch signaling pathway	3.27E-06
14	GO:0046533	negative regulation of photoreceptor cell differentiation	3.33E-06
14	GO:0050671	positive regulation of lymphocyte proliferation	3.33E-06
14	GO:0060907	positive regulation of macrophage cytokine production	3.33E-06
14	GO:0070723	response to cholesterol	3.33E-06
14	GO:0043066	negative regulation of apoptosis	4.38E-06
14	GO:0030326	embryonic limb morphogenesis	4.64E-06
14	GO:0048701	embryonic cranial skeleton morphogenesis	5.60E-06
14	GO:0046330	positive regulation of JNK cascade	6.58E-06
14	GO:0045892	negative regulation of cellular transcription, DNA-dependent	6.80E-06
14	GO:0007507	heart development	8.20E-06
14	GO:0002238	response to molecule of fungal origin	9.97E-06
14	GO:0002755	MyD88-dependent toll-like receptor signaling pathway	9.97E-06
14	GO:0051491	positive regulation of filopodium assembly	9.97E-06
15	GO:0006468	protein amino acid phosphorylation	5.74E-24
15	GO:0007169	transmembrane receptor protein tyrosine kinase signaling pathway	5.68E-09
15	GO:0043066	negative regulation of apoptosis	4.23E-07
15	GO:0007026	negative regulation of microtubule depolymerization	2.77E-06
15	GO:0018107	peptidyl-threonine phosphorylation	3.51E-06
15	GO:0018105	peptidyl-serine phosphorylation	7.83E-06
15	GO:0007242	intracellular signaling cascade	8.55E-06
16	GO:0006810	transport	4.54E-43
16	GO:0006811	ion transport	4.92E-41
16	GO:0007268	synaptic transmission	3.84E-24
16	GO:0007214	gamma-aminobutyric acid signaling pathway	8.54E-22
16	GO:0006816	calcium ion transport	3.99E-18
16	GO:0042391	regulation of membrane potential	3.73E-14
16	GO:0006821	chloride transport	7.40E-14
16	GO:0060079	regulation of excitatory postsynaptic membrane potential	4.81E-12
16	GO:0006874	cellular calcium ion homeostasis	4.24E-10
16	GO:0007612	learning	8.72E-10
16	GO:0007613	memory	8.72E-10
16	GO:0035249	synaptic transmission, glutamatergic	8.40E-09
16	GO:0048167	regulation of synaptic plasticity	1.60E-08
16	GO:0019228	regulation of action potential in neuron	2.63E-08
16	GO:0001964	startle response	3.57E-08
16	GO:0007269	neurotransmitter secretion	6.95E-08
16	GO:0007628	adult walking behavior	3.14E-07
16	GO:0006813	potassium ion transport	3.77E-07
16	GO:0001662	behavioral fear response	1.86E-06
16	GO:0050808	synapse organization	2.95E-06
16	GO:0030534	adult behavior	3.62E-06
16	GO:0048169	regulation of long-term neuronal synaptic plasticity	3.62E-06
16	GO:0006887	exocytosis	5.72E-06
16	GO:0019233	sensory perception of pain	8.54E-06

P-values were computed using one-sided Fisher exact test and the enriched terms with $p<1\times10^{-5}$ were listed in the table.

Figure S1. Hierarchical clustering result of the membership matrix obtained from prion network dataset. Based on the result, 16 modules indicated by blue squares were identified.

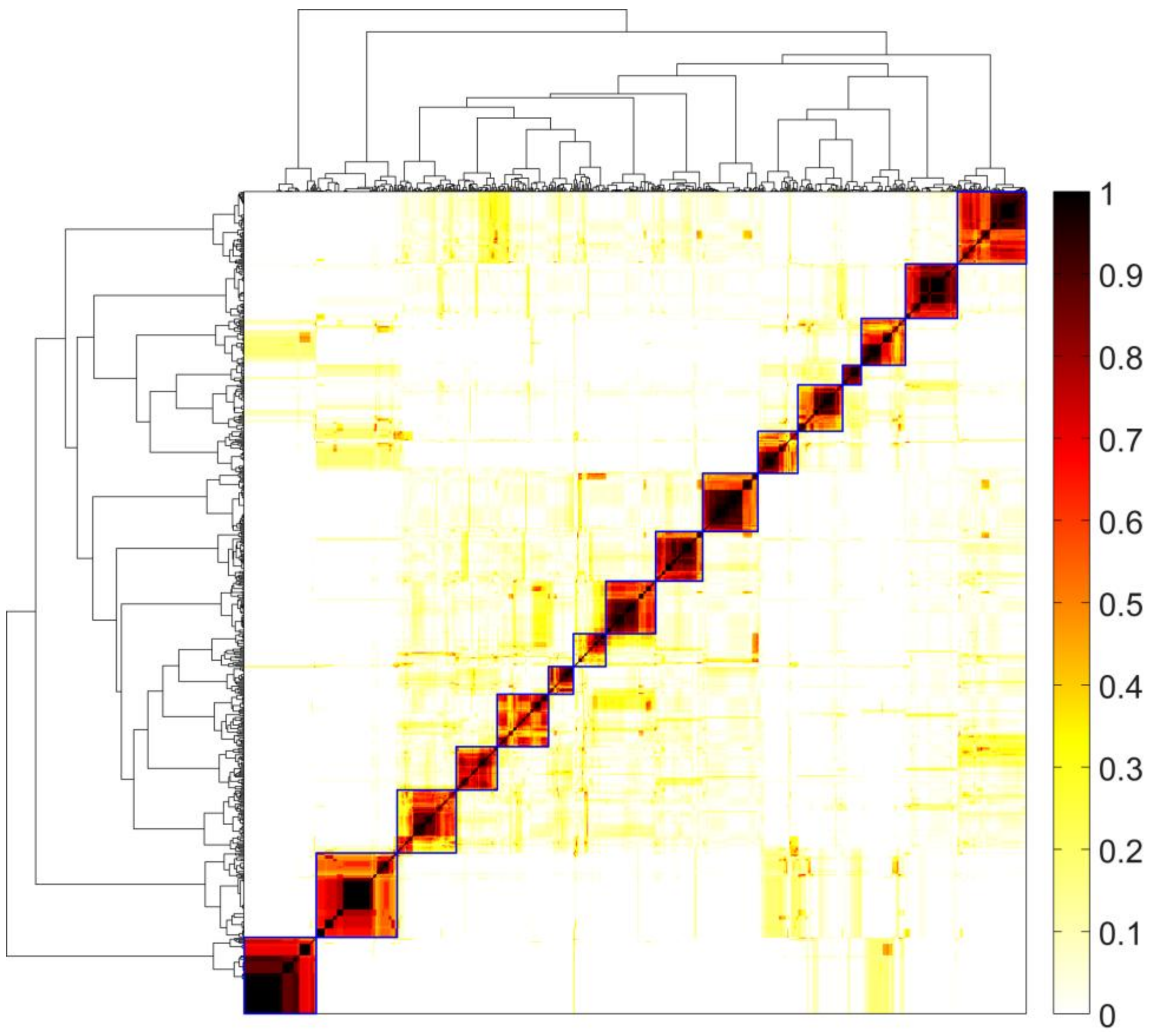

Figure S2. PrP^{Sc} accumulation subnetwork. Genes in the network correspond to SSIM module 1 and 3 were indicated by blue and black gene symbols and node borders, respectively. SSIM module 1 and 3 effectively identified core processes responsible for PrP^{Sc} accumulation, lysosomal proteolysis and lipid (cholesterol and sphingolipid) metabolism, respectively.

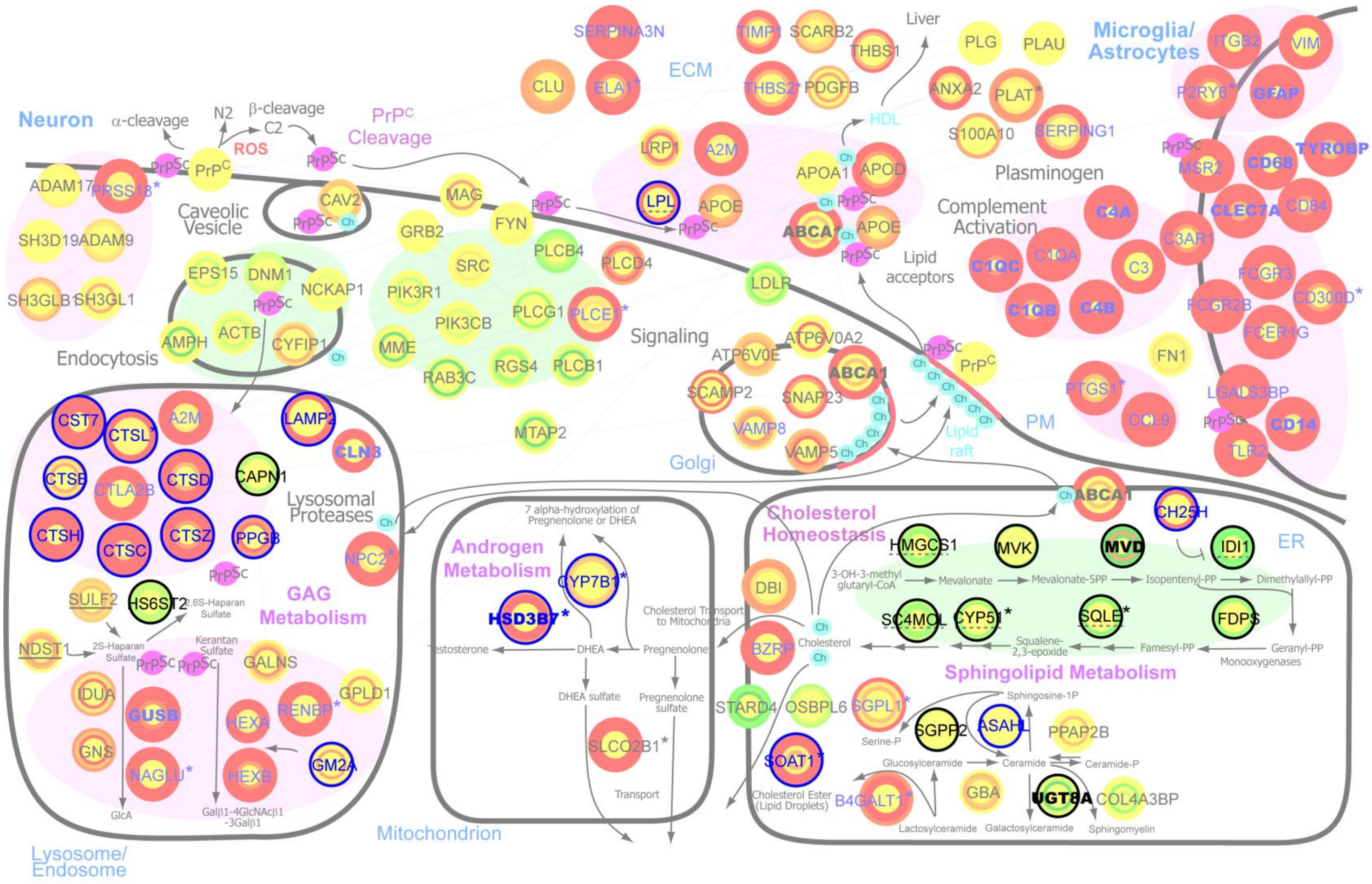

Figure S2. Microglial activation subnetwork. Genes correspond to SSIM module 2, 11, 13 and 14 were indicated by black, purple, green and blue gene symbols and node borders, respectively. They are associated with leukocyte migration (SSIM module 2, black), chemokine interactions (SSIM module 11, purple), complement and antigen presentation (SSIM module 13, green) and leukocyte activation (SSIM module 14, blue). It means that SSIM modules can stratify genes into functional groups responsible for the activation of microglial cells.

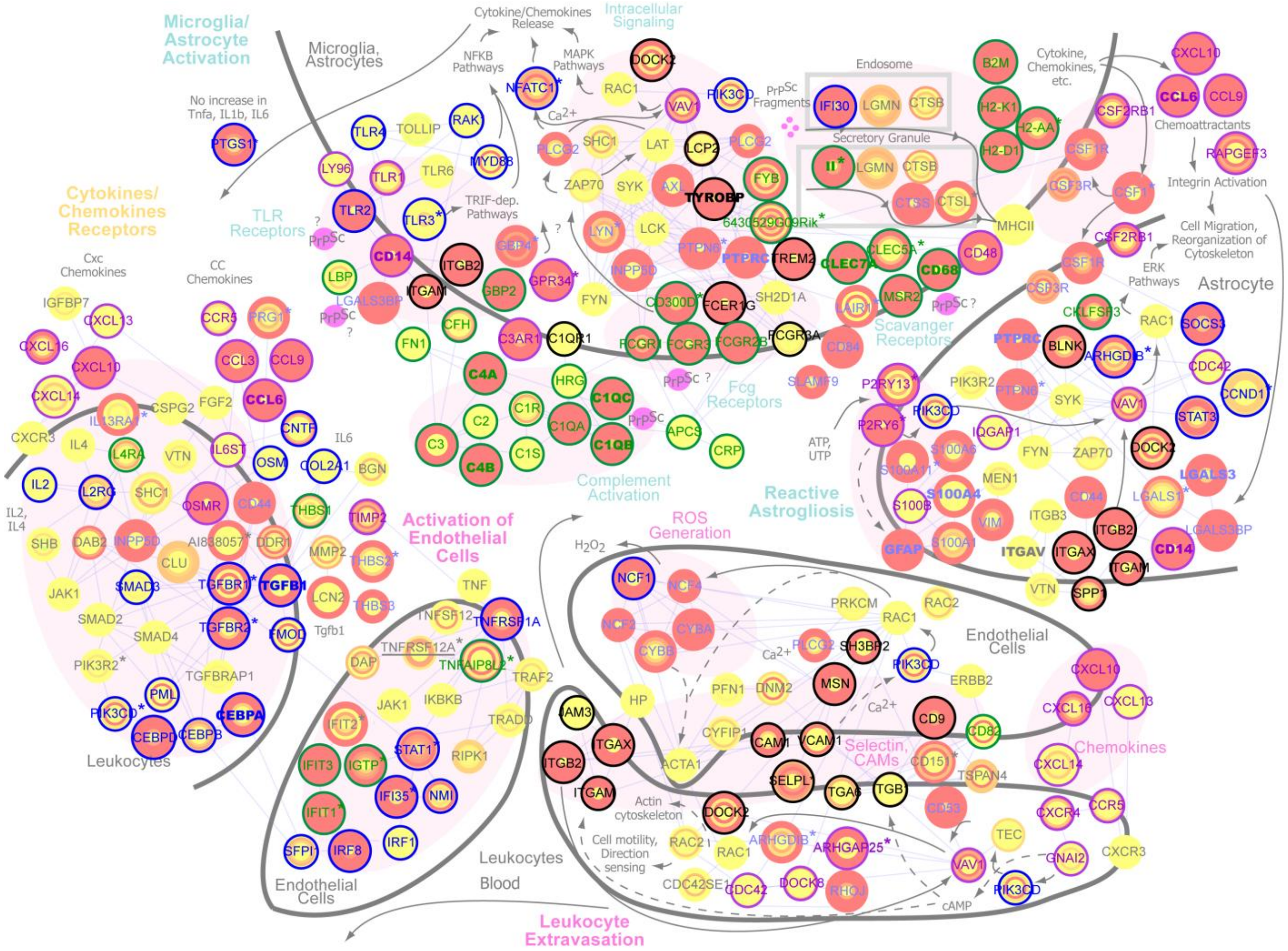

Figure S3. Neuronal cell death subnetwork. SSIM module 9 described by blue gene symbols and node borders represents caspase activation and mitochondrial disruption, main cellular processes of apoptosis.

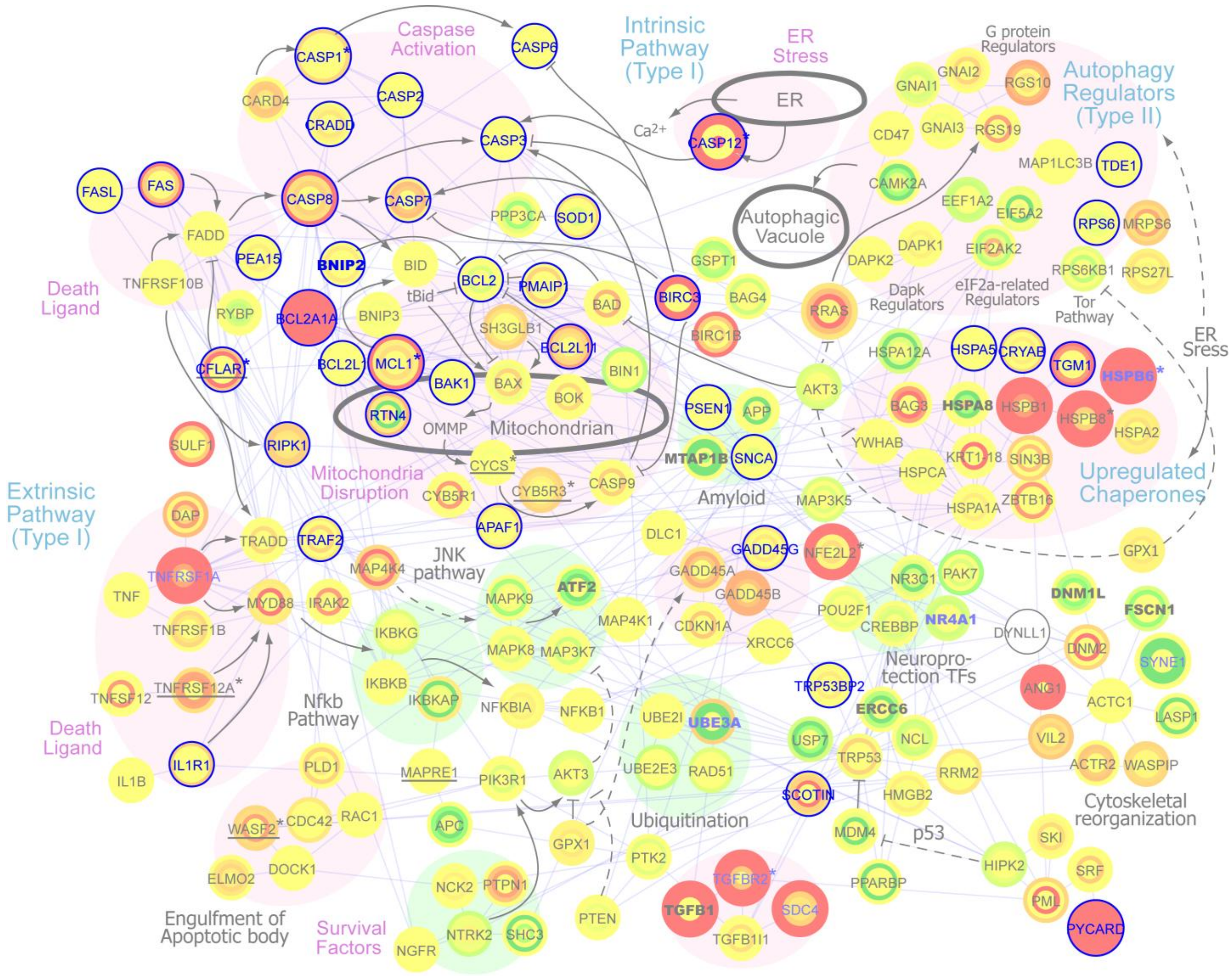

Figure S4. Synaptic degeneration subnetwork. SSIM module 6, 15 and 16, which are associated with transcription (histone modification)-related functions, intracellular signal transduction and ion/neurotransmitter influx/efflux-related functions, were depicted by black, red and blue gene symbols and node borders, respectively.

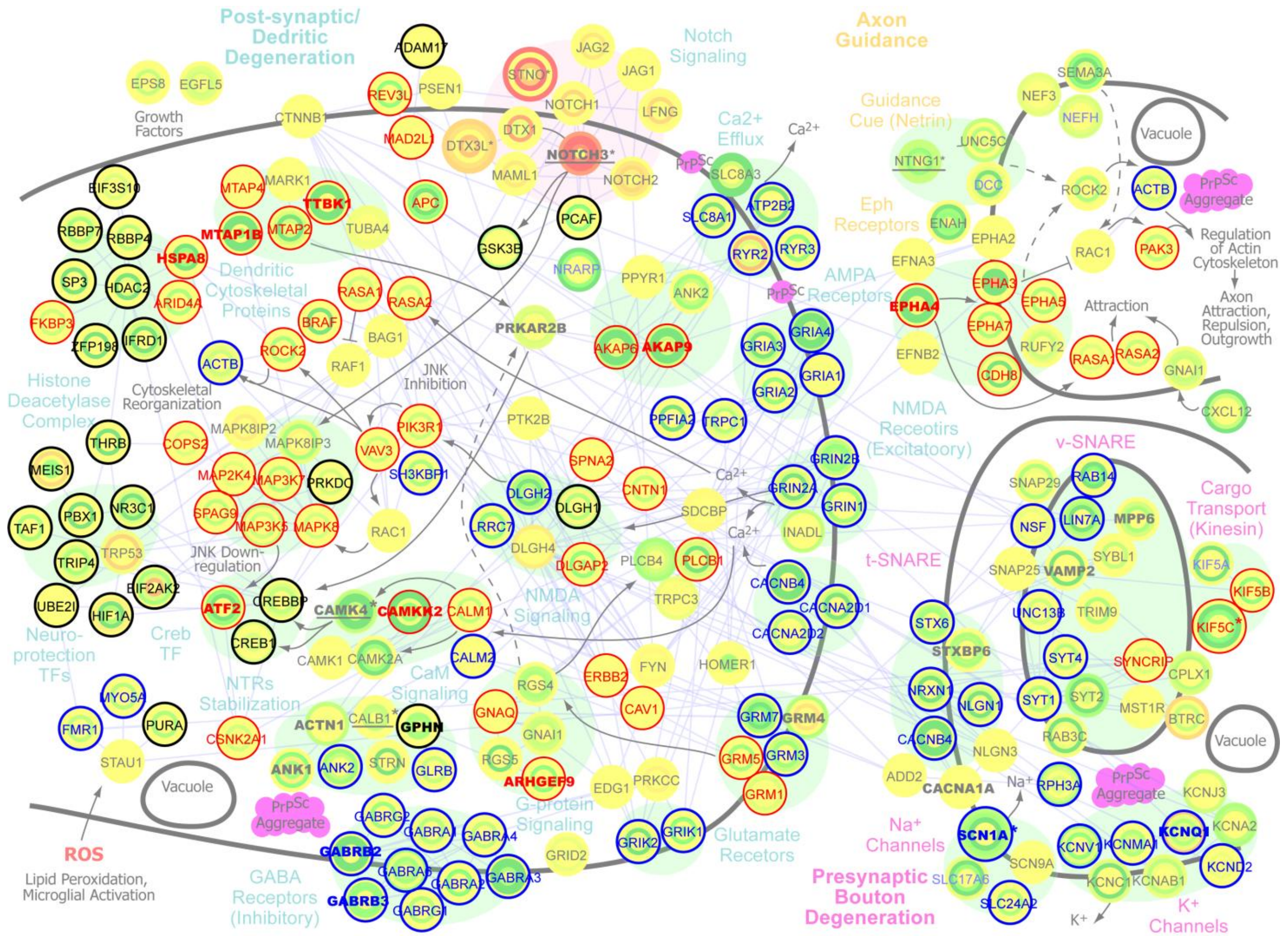

References

1. Hwang D, Lee IY, Yoo H, Gehlenborg N, Cho JH, Petritis B, Baxter D, Pitstick R, Young R, Spicer D, et al: **A systems approach to prion disease.** *Mol Syst Biol* 2009, **5:**252.
2. Gehlenborg N, Hwang D, Lee IY, Yoo H, Baxter D, Petritis B, Pitstick R, Marzolf B, Dearmond SJ, Carlson GA, Hood L: **The Prion Disease Database: a comprehensive transcriptome resource for systems biology research in prion diseases.** *Database (Oxford)* 2009, **2009:**bap011.
